# Local Overexpression of V1a-Vasopressin Receptor Enhances Regeneration in Tumor Necrosis Factor-Induced Muscle Atrophy

**DOI:** 10.1155/2014/235426

**Published:** 2014-05-20

**Authors:** Alessandra Costa, Angelica Toschi, Ivana Murfuni, Laura Pelosi, Gigliola Sica, Sergio Adamo, Bianca Maria Scicchitano

**Affiliations:** ^1^DAHFMO Unit of Histology and Medical Embryology, Interuniversity Institute of Myology, Sapienza University of Rome, Via A. Scarpa 16, 00161 Rome, Italy; ^2^Institute of Histology and Embryology, Catholic University School of Medicine, L.go F. Vito, 1, 00168 Rome, Italy

## Abstract

Skeletal muscle atrophy occurs during disuse and aging, or as a consequence of chronic diseases such as cancer and diabetes. It is characterized by progressive loss of muscle tissue due to hypotrophic changes, degeneration, and an inability of the regeneration machinery to replace damaged myofibers. Tumor necrosis factor (TNF) is a proinflammatory cytokine known to mediate muscle atrophy in many chronic diseases and to inhibit skeletal muscle regeneration. In this study, we investigated the role of Arg-vasopressin-(AVP-)dependent pathways in muscles in which atrophy was induced by local overexpression of TNF. AVP is a potent myogenesis-promoting factor and is able to enhance skeletal muscle regeneration by stimulating Ca^2+^/calmodulin-dependent kinase and calcineurin signaling. We performed morphological and molecular analyses and demonstrated that local over-expression of the AVP receptor V1a enhances regeneration of atrophic muscle. By upregulating the regeneration/differentiation markers, modulating the inflammatory response, and attenuating fibrogenesis, the stimulation of AVP-dependent pathways creates a favourable environment for efficient and sustained muscle regeneration and repair even in the presence of elevated levels of TNF. This study highlights a novel *in vivo* role for AVP-dependent pathways, which may represent an interesting strategy to counteract muscle decline in aging or in muscular pathologies.

## 1. Introduction

Skeletal muscle is a dynamic tissue capable of extensive regeneration in response to injury. Nevertheless, regeneration may be hindered in the case of aging, extended injury, or pathological conditions, leading to functional impairment. Although the general mechanisms underlying muscle regeneration have been identified, little is known about the factors limiting efficient repair in pathological muscle. Reduction in the number of satellite cells, poor recruitment of circulating stem cells within the damaged area, chronic inflammation, and formation of fibrotic tissue represent important factors contributing to limited or impaired regeneration. Inflammation is an important phase in the muscle regeneration process [1, 2]. Cytokines expressed during the early phase of inflammatory responses, such as interferon-*γ* (IFN-*γ*), interleukin-1*β* (IL-1*β*), and TNF, drive the differentiation and activation of macrophages towards the M1 phenotype. These classically activated M1 macrophages represent a proinflammatory population of cells capable of amplifying and perpetuating the inflammatory response [1]. After M1 macrophages reach their peak concentration, they are replaced by a population of alternatively activated M2 macrophages, which are activated by anti-inflammatory cytokines such as interleukin-4 (IL-4), interleukin-10 (IL-10), express CD-163 [1, 3, 4], and attenuate the inflammatory response and promote tissue repair. Perturbations in the spatial distribution of inflammatory cells, changes in the type and magnitude of the inflammatory cell infiltrate, and disrupted temporal sequence, result in a persistent rather than resolved inflammatory phase [5] and functional impairment of skeletal muscle [6].

The nuclear-factor-kappa B (NF-*κ*B) transcription factor is one of the most important molecules, which has been linked to the loss of skeletal muscle mass in various physiopathological conditions. Activation of NF-*κ*B in skeletal muscle leads to degradation of muscle specific proteins, inflammation, and fibrosis and blocks myofiber regeneration after injury/atrophy [7, 8]. Interestingly, NF-*κ*B regulates the expression of a number of inflammatory molecules including proinflammatory cytokines such as TNF, which is also a potent activator of NF-*κ*B, thus establishing a positive feedback loop leading to muscular abnormalities [9]. Although the autoactivation of NF-*κ*B requires proteolytic processing of NF-*κ*B and I*κ*B family of proteins by the ubiquitin-proteasome system [9, 10], NF-*κ*B can induce the expression of FoxO transcription factors by modulating the Akt signaling pathways [11]. Nuclear translocation and activation of FoxO transcription factors results in the upregulation of atrogin-1/MAFbx and MuRF1, thus promoting atrophy and muscle loss [12–14].

In this study, we examined the effects of local V1a-vasopressin receptor overexpression on TNF-induced muscle atrophy. The neurohypophyseal nonapeptide Arg^8^-vasopressin (AVP), oxytocin (OT), and related peptides constitute a family of positive regulators of terminal differentiation in myogenic cell lines (L5 and L6) and primary satellite cells [15–17]. V1aR is the only vasopressin receptor expressed in skeletal muscle [18–20]. Previously, we demonstrated that V1aR expression is modulated during skeletal muscle regeneration, and that AVP-V1aR signaling is a powerful enhancer of muscle regeneration through mechanisms involving calcineurin A (CnA), GATA2, MEF2, and IL-4 [21]. There is now extensive evidence showing that the AVP system is impaired in several neuromuscular diseases, such as amyotrophic lateral sclerosis and multiple sclerosis [22, 23], suggesting that AVP may act as a physiologic factor in skeletal muscle homeostasis.

Here we demonstrate that local V1aR overexpression enhances regeneration in TNF-induced muscle atrophy. By modulating the inflammatory response, attenuating fibrogenesis, and upregulating the regeneration/differentiation markers, the stimulation of AVP-dependent pathways creates a favourable environment for sustained and efficient muscle regeneration and repair even in the presence of elevated levels of TNF. In addition, we demonstrate that the positive effect of V1aR overexpression in muscle homeostasis involves Akt-mediated inhibition of FoxO transcription factors.

The findings of this research are expected to lead to the identification of new pharmacological or gene therapy targets that may delay the progression of muscular wasting associated with numerous myopathies.

## 2. Materials and Methods

### 2.1. Animals

C57 transgenic desmin/nls-lacZ mice used in this study bear a transgene in which the 1-*κ*B DNA 5′ regulatory sequence of the desmin gene is linked to a reporter gene coding for* Escherichia coli*-*β*-galactosidase [24]. The desmin/nls-lacZ transgene labels muscle cells in which the desmin synthesis program has commenced [25]. Mice were treated according to the guidelines of the Institutional Animal Care and Use Committee. They were housed in a temperature-controlled (22 ± 2°C) and humidity-controlled (60 ± 5%) room regulated to provide a 12 h light, 12 h dark cycle. Mice were allowed to feed and drink* ad libitum*. Animals were anesthetized with an intraperitoneal injection of Avertin A (a mix of tribromoethanol and 2-methyl-2-butanol diluted in physiological solution) before gene delivery by electroporation or muscle damage. Injury of mock-, V1aR-, TNF-, and V1aR+TNF-transfected* Tibialis anterior* (TA) muscles of 2-month-old mice was induced along the entire length of the muscle with a total of four cardiotoxin (CTX) injections (5 *μ*L of 10 *μ*M CTX per injection). In our previous study, regeneration was fully active at 7 days after injury [21].

Therefore, mice were sacrificed 7 days after injury and the regeneration process in the injured muscle was examined by assessing the number and size of regenerating fibers as previously described [21].

### 2.2. Plasmid Construction

The MLC-Myc-V1aR plasmid was derived from MLC-Myc and PCD3-V1aR (kindly provided by Prof. S.J. Lolait, University of Bristol, UK) expression vectors, as previously described by Toschi et al. [21]. To induce expression of the secreted form of murine TNF-*α*, we used the construct pBabe-mTNF-*α* (kindly provided by Dr. Gokhan Hotamisligil, Harvard University, Boston, MA) under control of the SV40 promoter. The SV40 promoter has been shown to be efficient for driving exogenous cDNA expression in skeletal muscle [26].

### 2.3. Gene Delivery by Electroporation

The TA in each mouse hind limb was injected with the indicated amount of cDNA: 20 *μ*g of MLC-Myc-V1aR (V1aR), or pcDNA3 (mock) [21], or pBabe-mTNF-*α* (TNF)m or 10 *μ*g of MLC-Myc-V1aR plus 10 *μ*g of pBabe-mTNF-*α* (V1aR+TNF), in combination with 5 *μ*g of pCMV-SNAP-GFP (kindly provided by Dr. Pozzan, University of Padua, Italy), as a marker of transfection efficiency. The electric pulses were delivered using 3 × 5 mm gene paddles electrodes (BTX, San Diego, CA) placed on either side of the muscle, as described by Donà et al. [27]. This protocol of gene delivery by electroporation guarantees stable DNA expression for more than four months. After electroporation, mice rapidly recovered and did not show any locomotor impairment or particular sign of pain or stress.

### 2.4. Histological and Histochemical Analysis

TA muscles from mock-, V1aR-, TNF or TNF+V1aR-transfected 7-8-week-old desmin/nls-lacZ mice were embedded in Jung tissue freezing medium (Leica, Wetzlar, Germany) and frozen in liquid nitrogen-cooled isopentane. Frozen sections (7 *μ*m) were obtained using a Leica cryostat. Sections were observed under the green activation filter of the Axioskop 2 plus system (Zeiss). For histological analysis, sections were stained with hematoxylin and eosin (H&E) using standard methods [28].

Esterase staining was adapted from Davis [29] as previously reported [30]. Cryosections of each muscle were incubated for 5 min in a staining solution containing 3 mg alpha-naphthyl acetate, 0.375 mL acetone, 6.25 mL 0.2 M sodium phosphate, and 0.4 mL of a solution containing equal volumes of 2% pararosaniline (Sigma-Aldrich) and 2% sodium nitrite. Photomicrographs were obtained using an Axioscop2 plus system equipped with an AxiocamHRc (Zeiss, Oberkochen, Germany) at 1300 × 1030 pixel resolution and analyzed using 10x NA 0.30 air objective lens or 20x NA 0.50 air objective lens.

The trichrome stain (Masson) kit (Sigma-Aldrich Procedure number HT15) was used for distinguishing collagen from muscle tissue. Tissue sections were treated with Bouin's solution to intensify the final coloration. Nuclei were stained with Weigert's iron hematoxylin, and the cytoplasm and muscle were stained with Biebrich scarlet-acid fuchsin. After treatment with phosphotungstic and phosphomolybdic acid, collagen was demonstrated by staining with aniline blue.

### 2.5. Immunofluorescence Analysis

Frozen sections were fixed in 4% paraformaldehyde for 10 min on ice, washed with PBS, incubated in PBS containing 1% BSA (Sigma-Aldrich) and 1 : 100 goat serum for 30 min at room temperature, and then incubated overnight at 4°C with the selected primary antibody at the appropriate dilution. The following antibody was used: mouse monoclonal antiembryonic MHC (Developmental Hybridoma-Bank number BF-G6, University of Iowa, Iowa City, IA). Samples were then washed with PBS containing 1% BSA and incubated for 1 h at room temperature with the appropriate secondary antibody, Alexafluor 568-conjugated anti-mouse (Molecular Probes, Eugene, OR, USA) 1 : 500 in 1% BSA. Nuclei were visualized with Hoechst 33342 (Sigma-Aldrich). The sections were mounted with Vectashield mounting medium (Vector Laboratories, Burlingame, CA) and examined in a Zeiss Axioplan (Zeiss, Thornwood, NY) fluorescence microscopy using a 10x and 20x objective lens.

### 2.6. Morphometric Analysis

Photomicrographs of regenerating muscle fibers (identified by morphological criteria, i.e., centrally located nuclei in H&E stained sections) were taken at standard resolution (1.300 × 1030 pixel) and analyzed using ImageJ, Scion Image software. For morphometric evaluation of fiber size, 200–1000 cross-sectioned fibers per sample were analyzed. Quantitative data were obtained from three independent experiments in triplicate. The values are expressed as mean ± SD.

### 2.7. Gene Expression Analysis

TA muscles were dissected, and total RNA extraction was performed with tissue lyser (QIAGEN) in TriRagent (Invitrogen, Carlsbad, CA), according to the manufacturer's protocol, and was reverse-transcribed using Moloney Murine Leukemia Virus Reverse Transcriptase (M-MLV RT; Invitrogen, Carlsbad, CA). cDNA preamplification was performed with TaqMan PreAmp Master Mix Kit (Applied Biosystems) according to the manufacturer's instructions [31–33] to accurately detect the expression pattern of the following cytokines: IL4, IL10, IL6, IL1*β*, and the chemokine ligand CCL2 and scavenger receptor CD163. Quantitative PCR was performed on an ABI PRISM 7500 SDS (Applied Biosystems, USA), using premade 6-carboxyfluorescein (FAM)-labeled TaqMan assay for: TNF (Mm00443258_m1), V1aR (Mm00444092_m1), CCL2 (Mm00441242-m1), IL1-*β* (Mm01336189_m1), IL6 (Mm0120733_m1), CD163 (Mm004744096_m1), IL10 (Mm00439616_m1), IL-4 (Mm00445260_m1) Pax7 (Mm00834082_m1), desmin (Mm00802455_m1), myogenin (Mm00446194_m1), atrogin-1 (Mm01207878_m1), and HPRT (Mm00446968_m1) as internal controls. Real-time PCR was performed using RNA preparations from three different animals for each group.

### 2.8. Immunoblotting Analysis

TA muscles were dissected, minced, and homogenized with RIPA buffer (20 mMTris/HCl pH 7.5, 2 mM EDTA, 2 mM EGTA, 0.25 M Sucrose, 5 mM DTT, 0,1% Triton X-100, 10 mM NaF, 200 *μ*M sodium orthovanadate) containing a protease inhibitor cocktail (Roche, Indianapolis, IN). Equal amounts of protein (20–30 *μ*g), determined by Pierce BCA Protein Assay Reagents, were separated by SDS PAGE and transferred electrophoretically to a hybond-C extra nitrocellulose membrane (Amersham Biosciences, Piscataway, NJ). Nonspecific binding was blocked in Tris-buffered saline with Tween 20 (TBST) containing 5% nonfat milk for 1 h at room temperature, and the membrane was then incubated overnight at 4°C in TBST containing primary antibodies. The following antibodies were used: mouse monoclonal anti-MHC (Developmental Hybridoma-Bank), monoclonal anti-NF*κ*B-p65 (C22B4), and monoclonal anti-phosphoNF*κ*B-p65 (Ser563) (93H1) (Cell Signaling), rabbit polyclonal anti-I*κ*B*α* (Santa Cruz Biotechnologies), polyclonal anti-phospho-Akt (Ser473), monoclonal anti-FoxO3a (75D8), and polyclonal anti-phospho-FoxO3a (Ser253) (Cell signaling). Monoclonal anti-tubulin-*α* (Sigma-Aldrich) or mouse monoclonal anti-GAPDH (Santa Cruz Biotechnologies) was used for normalization, as indicated. Blots were washed in TBST and then incubated with the appropriate secondary antibodies, goat anti-mouse, or anti-rabbit HRP-conjugated (Bio-Rad Laboratories, Hercules, CA) in TBST containing 1% nonfat milk. Blots were extensively washed and the antibody binding was detected by means of Super Signal West Pico Chemiluminescent Substrate (Pierce, Rockford, IL).

### 2.9. Statistical Analysis

The Student's *t*-test was used throughout this paper for statistical analyses.

## 3. Results

### 3.1. Local V1aR Overexpression Counteracts the Negative Effects of TNF on Muscle Regeneration

**Figure 1:**
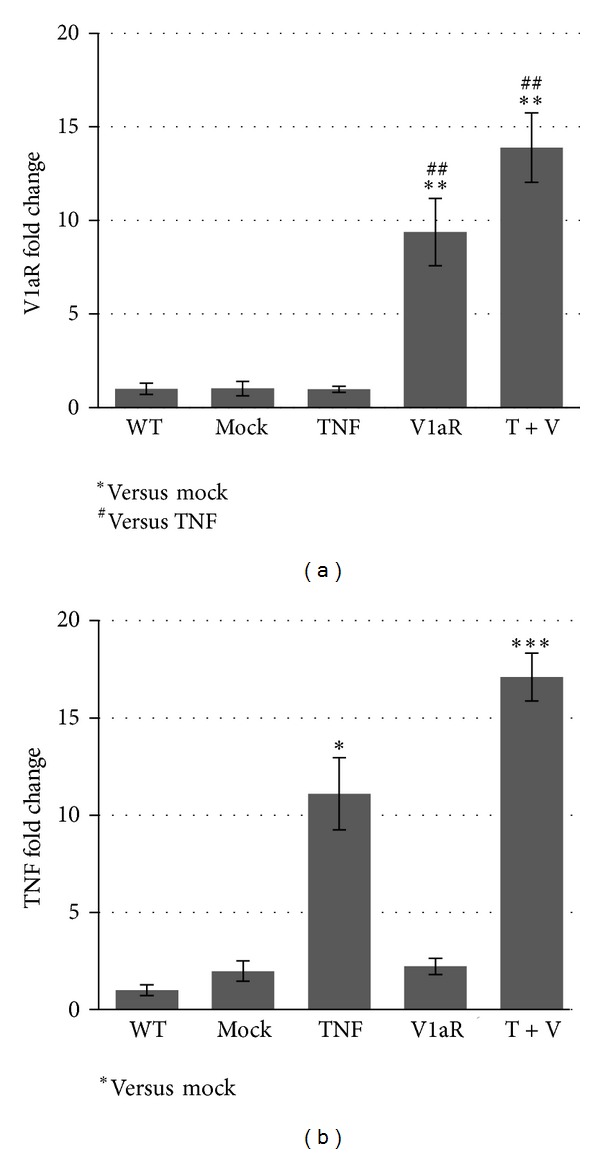
Expression of the transfected cDNA constructs is increased in electroporated muscles. Real-time PCR analysis of V1aR (a) and TNF (b) was performed on RNA extracts obtained from WT, mock-, V1aR-, TNF-, and V1aR+TNF-transfected TA muscles to verify the transfection efficiency after electroporation. The expression of V1aR and TNF is not significantly different when comparing V1aR versus V1aR+TNF and TNF versus V1aR+TNF samples, respectively (Figures 1(a) and 1(b)). Values represent the average three independent experiments. *P < 0.05; **P < 0.01; ***P < 0.001 by Student's *t*-test.

Our previous data demonstrated that muscle specific overexpression of the AVP receptor V1a enhances skeletal muscle regeneration after CTX induced damage, by stimulating satellite cell activation and increasing the expression of differentiation markers [21]. We also demonstrated that TNF administration is sufficient to induce inhibition of muscle regeneration by activating a nonapoptotic, caspase-dependent process, ultimately leading to muscle wasting [34]. Therefore, in this study we investigated whether local overexpression of V1aR protects the muscle from the effects of high TNF levels. To this end, we overexpressed the myosin-light-chain (MLC)-myc-V1a AVP receptor construct [21] alone or in combination with the TNF construct [21, 35] in TA muscles, by means of gene delivery by electroporation. Controls consisted of mock-transfected samples (i.e., muscles transfected with pcDNA3) and, when indicated, in nonelectroporated muscles (WT). In order to assess the efficacy of the transfections, we used real-time PCR analysis, to verify the V1aR and TNF expression levels in muscle extracts one week after electroporation (Figures 1(a) and 1(b)). V1aR expression was strongly upregulated in TA muscles electroporated with the V1aR construct, both in the presence and in the absence of TNF, demonstrating that TNF did not interfere with the electroporation efficiency or with the forced expression of V1aR (Figure 1(a)). As expected, TNF expression was high in samples electroporated with the TNF construct alone or in combination with V1aR (Figure 1(b)). The expression of V1aR and TNF did not differ significantly between the V1aR and V1aR+TNF samples (Figures 1(a) and 1(b)).

**Figure 2:**
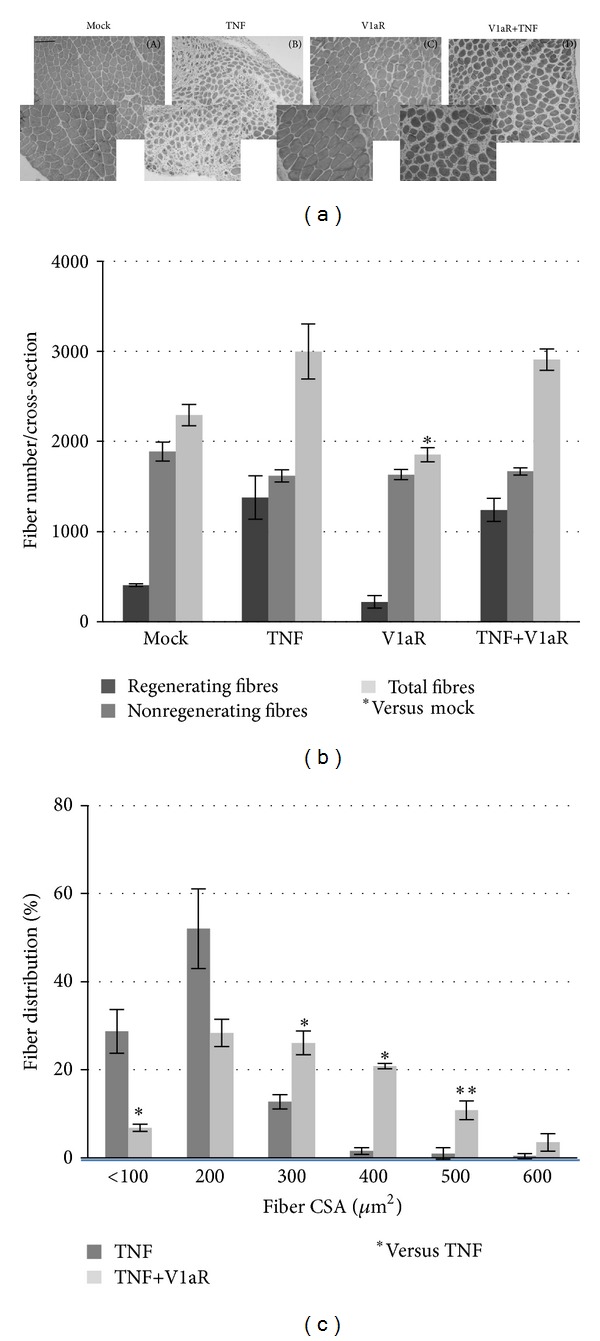
Local V1aR overexpression in atrophic muscle enhances the regeneration process. (a) Cross-sections of mock (A), TNF- (B), V1aR- (C), and TNF+V1aR- (D) transfected TA were stained with H&E one week after electroporation. Scale bar = 50 *μ*m. Original magnification 10x and insert magnification 20x. (b) Diagram showing the number of regenerating, nonregenerating, and total fibers per cross-sectional area. *P < 0.05. (c) Morphometric analysis shows that in muscles overexpressing V1aR and TNF together the mean fiber cross sectional area (CSA) is bigger compared with samples overexpressing TNF alone. *P < 0.05; **P < 0.01 by Student's *t*-test.

Morphological analysis showed that 1 week after electroporation, local overexpression of TNF (Figure 2(a), panel (B)) caused an accumulation of mononucleated infiltrating cells, compared with mock-transfected and V1aR overexpressing muscle alone (Figure 2(a), panels (A) and (C) resp.). In addition, a regeneration response occurred in the muscle rendered atrophic by TNF overexpression, as demonstrated by the presence of central nucleated fibers in TNF overexpressing muscle (Figure 2(b)). By contrast, cotransfection of V1aR and TNF did not significantly modify the number of regenerating fibers compared with TNF transfection alone. It did, however, affect the fiber size distribution by favoring the accumulation of larger fibers, as shown in Figure 2(c). It is noteworthy that the overexpression of V1aR induced a slight, yet significant increase in the fiber cross-sectional area compared with mock-transfected muscle (15% increase, *P* < 0.001) (data not shown). In conclusion, V1aR overexpression leads to more efficient regeneration than that observed in muscles overexpressing TNF alone.

### 3.2. Muscle V1aR Overexpression Modulates the Inflammatory Response and Attenuates the Fibrosis Induced by High Levels of TNF

**Figure 3:**
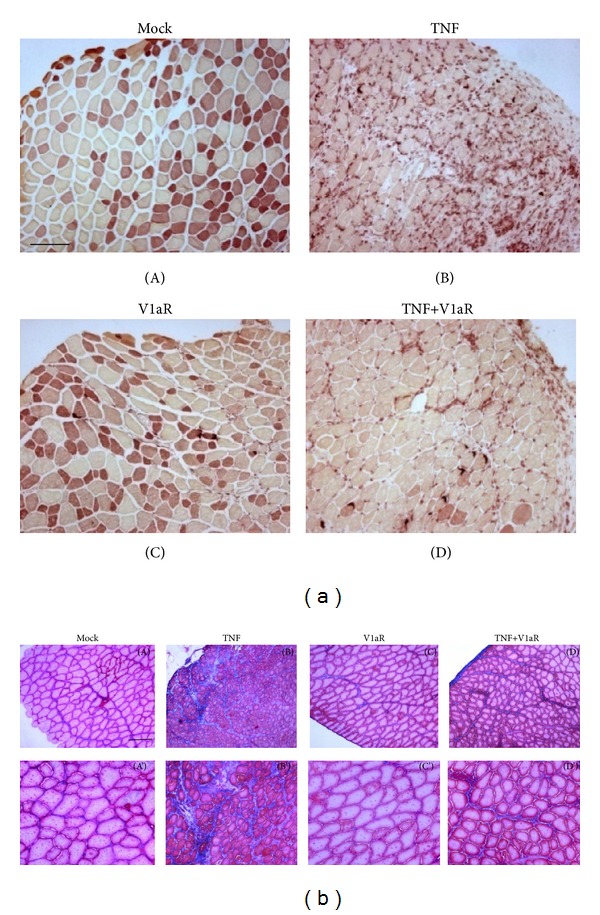
V1aR overexpression attenuates inflammation and fibrosis induced by high levels of TNF. (a) Nonspecific esterase staining of TA cross-sections, performed one week after electroporation. The figure highlights the massive presence of macrophages in muscle overexpressing TNF (B) and a reduced esterase activity in muscle overexpressing both TNF and V1aR (D). (Scale bar = 50 *μ*m, magnification 10x.) (b) Masson's trichrome stain of TA cross-sections, one week after electroporation, demonstrates less extensive fibrosis in muscle overexpressing TNF+V1aR (D and D'), compared with muscle overexpressing TNF alone (B and B'). (Scale bar = 50 *μ*m, magnification 10x, (A)–(D); 20x, (A')–(D').

TNF is a proinflammatory cytokine capable of activating macrophages, thereby inducing the production of other proinflammatory cytokines and perpetuating the inflammatory response. To verify whether the enhanced regenerative response in V1aR overexpressing muscle is associated with a modulation of inflammation, we examined the presence of macrophages by esterase staining.  Figure 3(a) shows a high number of esterase-positive, macrophages in TNF over-xpressing muscles, whereas the concomitant overexpression of V1aR significantly attenuated their number.

Efficient muscle repair is accompanied by and/or requires the migration and proliferation of fibroblasts needed to produce additional extracellular matrix (ECM) components acting as a scaffold for regenerating myofibers [3, 36, 37] and substituting the basement membrane and ECM components degraded during the inflammatory phases [38–40]. However, if inflammatory cell infiltration and fibroblast activation persist, an aberrant tissue repair response will produce a nonfunctional mass of fibrotic tissue [41]. In order to visualize the extent of fibrosis, we performed Masson's trichrome staining under the indicated experimental conditions.  Figure 3(b) shows the normal presence of connective tissue in mock muscle and in muscle overexpressing V1aR, while muscles expressing TNF display increased amounts of ECM in skeletal muscle tissue. In the presence of high TNF and V1aR levels, the extent of fibrosis was significantly attenuated in comparison with muscle overexpressing TNF alone. Taken together, these data demonstrate that V1aR overexpression leads to a more efficient regeneration process, characterized by attenuated inflammatory response and reduced fibrosis.

### 3.3. V1aR Overexpression Modulates the Molecular Mechanisms Involved in the Inflammatory Response

**Figure 4:**
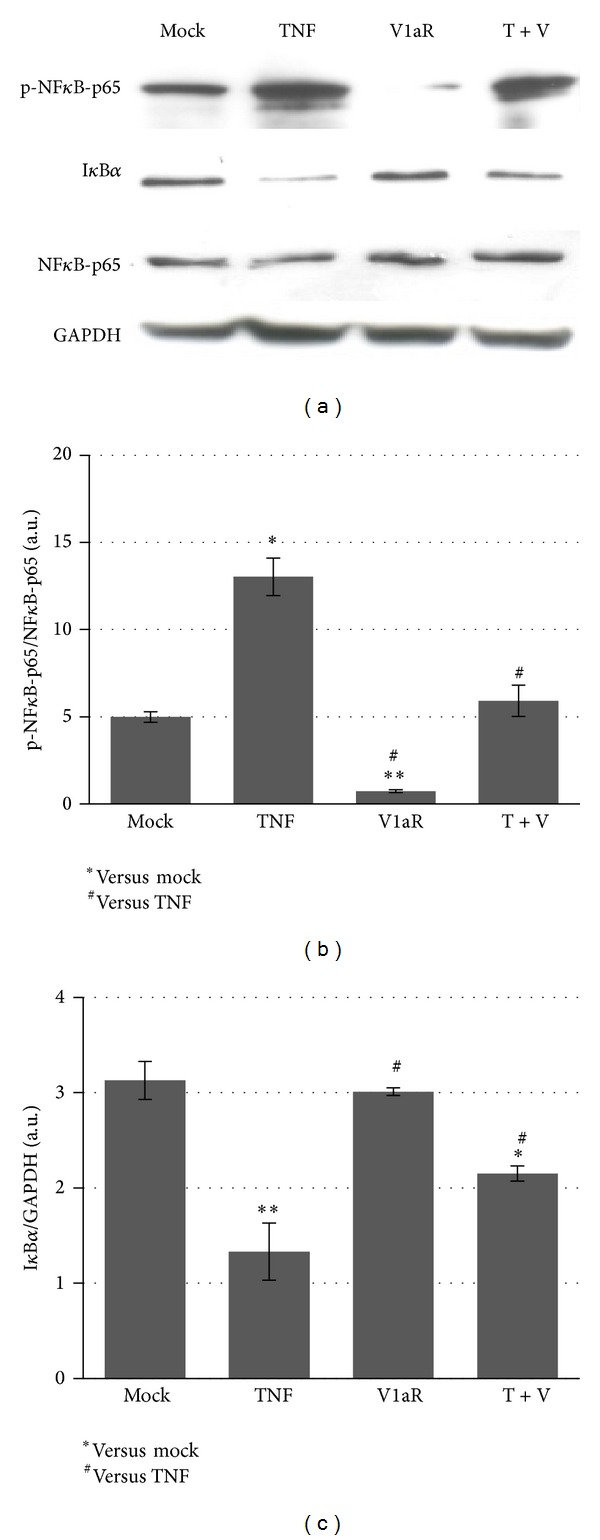
Downregulation of NF-*κ*B pathway in muscle over-expressing V1aR. (a) Western blot analysis revealed that TNF overexpression promotes downregulation of I*κ*B*α* with concomitant upregulation of the phosphorylated form of NF*κ*B (p-NF-*κ*B-p65) expression. By contrast, p-NF-*κ*B-p65 expression was significantly reduced in muscle overexpressing TNF and V1aR together compared with TNF alone. The expression of native NF-*κ*B-p65 does not reveal significant differences between the various samples. (b, c) Densitometric analysis of two independent experiments for phospho-NF-*κ*B-p65 versus native NF-*κ*B-p65 (b) and I*κ*B*α* versus GAPDH (c) expression. ^∗#^ *P* < 0.05; **P < 0.01 by Student's *t*-test.

It has been demonstrated that NF-*κ*B is one of the central players of the inflammatory system [2, 42] and that TNF, along with other proinflammatory cytokines, stimulates the NF-*κ*B signaling pathway, promoting muscle catabolism [5, 7]. Western blot and densitometric analysis (Figures 4(a), 4(b), and 4(c)) revealed that TNF overexpression promotes a strong upregulation of phospho-NF-*κ*B expression and a concomitant downregulation of the NF-*κ*B inhibitory factor, namely, I*κ*B*α*. By contrast, phospho-NF-*κ*B expression was significantly reduced in muscle overexpressing both TNF and V1aR, demonstrating that V1aR overexpression attenuates the effects of TNF on inflammation.

### 3.4. Inflammatory Cytokine Production Is Modulated in V1aR Overexpressing Muscle

**Figure 5:**
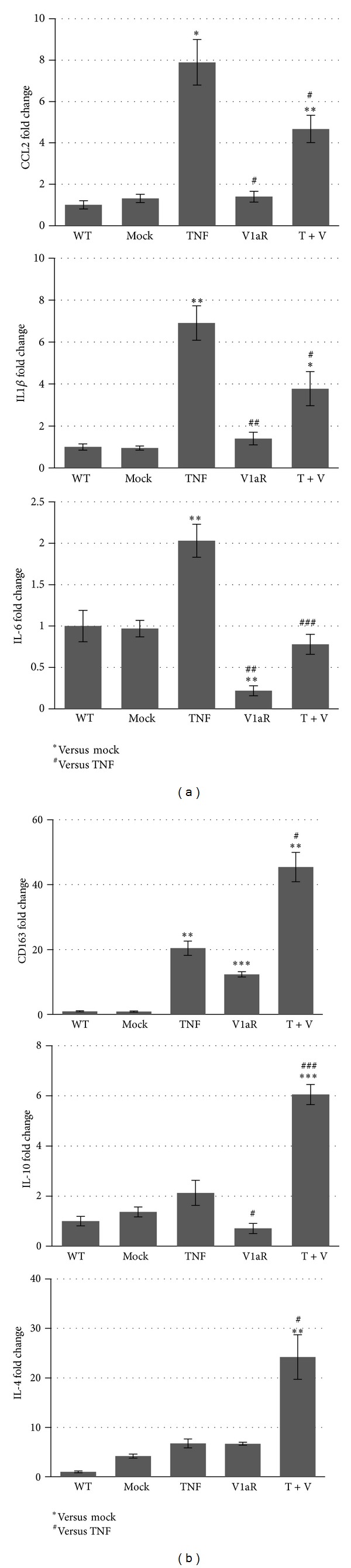
V1aR overexpression directs inflammation towards the repair phase. (a) Real-time PCR analysis for CCL2, IL-1*β*, and IL-6. These chemokines and cytokines typical of the first proinflammatory phase were highly transcribed in muscle overexpressing TNF alone, while the coexpression of V1aR significantly reduced their expression. By contrast, the expression levels of the anti-inflammatory cytokines CD163, IL-10, and IL-4 (b) were strongly induced in muscle overexpressing V1aR and TNF together. (b) ^∗, #^ *P* < 0.05; ^∗∗, ##^ *P* < 0.01; ^∗∗∗, ###^ *P* < 0.001 by Student's *t*-test.

To gain further insight into the mechanism by which V1aR modulates the resolution of inflammation and contributes to muscle repair, we performed real-time PCR, to analyse the expression patterns of specific cytokines and chemokines secreted by M1 macrophages, a proinflammatory cell population capable of perpetuating the inflammatory response [3].  Figure 5(a) demonstrates that the expression of CCL2, IL1*β* and IL6 was strongly upregulated in the presence of high levels of TNF compared with mock- and V1aR-transfected muscles. TNF overexpression was also accompanied by a slight increase in CD163 expression. By contrast, the overexpression of V1aR, in TNF expressed muscle, significantly reduced the expression of the proinflammatory cytokines. Interestingly, the reduction in M1 macrophages, promoted by V1aR overexpression, was associated with an upregulation of M2 macrophage markers such as CD163, IL-10, and IL-4 (Figure 5(b)). Notably, CD163 is a macrophage-specific receptor for complexes of hemoglobin and haptoglobin, and ligation of CD163 can contribute to the regulation of macrophage phenotype by increasing the expression of anti-inflammatory cytokines, especially IL-10 [43]. IL-4 is involved in muscle regeneration by promoting satellite cell fusion and differentiation [44–46], and we have previously demonstrated that stimulation of AVP signaling induces calcineurin-dependent expression of IL-4 in muscle cells. These results demonstrate that a high level of V1aR accelerates the resolution of TNF-dependent inflammation, modulating the muscle milieu by favoring the shift from M1 to M2 macrophage phenotype, thereby stimulating the secretion of anti-inflammatory cytokines and thus promoting regeneration.

### 3.5. Stimulation of AVP Signaling Modifies the Effects of TNF on Myogenesis and Promotes Muscle Maturation

**Figure 6:**
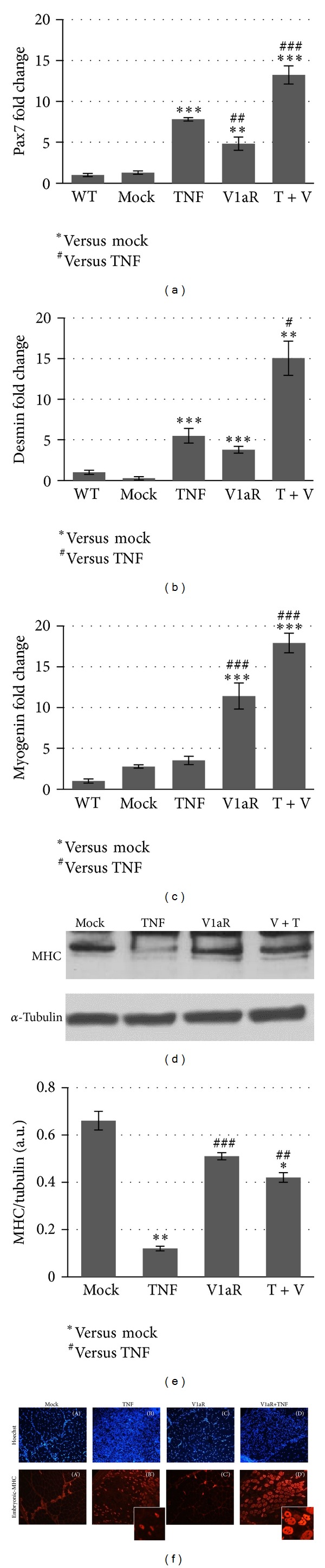
V1aR overexpression induces muscle regeneration even in the presence of TNF. Real-time PCR analysis shows that forced expression of TNF significantly increases the expression of Pax7 (a) and desmin (b) compared with mock-transfected samples. The expression level of these markers is even higher in muscle overexpressing TNF and V1aR together, while only a slight, yet significant, increase is observed in V1aR-transfected muscle. Interestingly, myogenin expression (c) does not significantly change in the presence of TNF, while it is strongly upregulated in V1aR overexpressing muscles, both in the presence and in the absence of TNF. (d) Western blot analysis shows a downregulation of MHC expression by TNF, while its expression increases in V1aR and V1aR+TNF overexpressing muscles. (e) Densitometric analysis of three independent experiments for MHC versus tubulin expression. *P < 0.05; **P < 0.01; ***P < 0.001 by Student's *t*-test. (f) Immunofluorescence analysis of embryonic MHC in TA cross-sections. Muscles overexpressing both TNF and V1aR display an increase in number and size of regenerating muscle fibers compared with TNF overexpressing muscle.

Regeneration consists of a sequence of phenomena including the activation of satellite cells, and their differentiation and fusion into fibers expressing muscle specific products. Therefore, we first analyzed the expression of Pax7 and desmin, which are relevant markers of activated and proliferating satellite cells, 1 week after TNF and/or V1aR electroporation in muscles. Real-time PCR analysis demonstrated that TNF overexpression greatly increased the levels of Pax7 and desmin expression, when compared with WT and mock-transfected muscle, whereas the transfection of V1aR alone induced lower but significant changes in the expression of these proteins (Figures 6(a) and 6(b)). By contrast, the combination of V1aR and TNF overexpression promoted a significant increase in Pax7 and desmin expression. We then analyzed the expression of molecular markers characteristic of the terminal phases of muscle differentiation, such as myogenin and MHC. It is noteworthy that TNF overexpression downregulated the expression levels of MHC (but not of myogenin) compared with mock-transfected samples (Figure 6(c) and densitometry Figure 6(e)). By contrast, V1aR overexpression counteracted the negative effect of TNF on muscle differentiation, stimulating myogenin and MHC expression (Figures 6(c) and 6(d) and densitometry Figure 6(e)). Moreover, immunofluorescence analysis for embryonic-myosin heavy chain (embryonic-MHC), a marker of regenerating myofibers, revealed a significant increase in the number of embryonic-MHC positive fibers in muscles overexpressing both TNF and V1aR, compared with control and V1aR-alone transfected samples (Figure 6(f)).

Taken together, these results suggest that TNF stimulates satellite cell activation and muscle regeneration but, as expected, impinges on the maturation process. By contrast, overexpression of V1aR counteracts the negative effects of TNF, stimulating muscle growth and maturation.

### 3.6. V1aR Counteracts the Effect of TNF on Protein Degradation by Stimulating PI3K/Akt/FoxO Signaling

**Figure 7:**
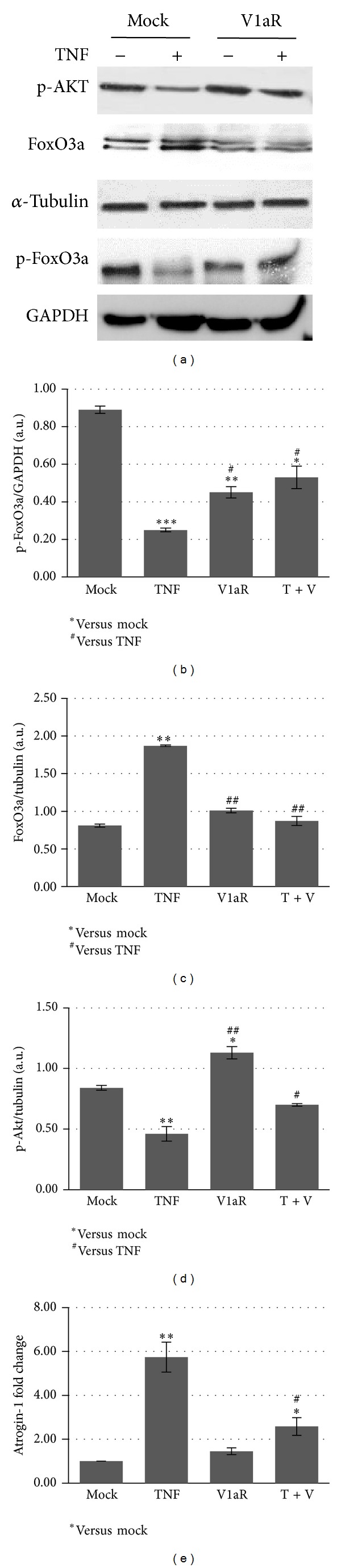
V1aR overexpression counteracts TNF-dependent protein degradation by stimulating the Akt pathway. (a) Western blots of phosphorylated Akt and native and phosphorylated FoxO3a expression demonstrate that in muscle overexpressing TNF, phospho-Akt and phospho-FoxO3a are downregulated, while the native Foxo3a is increased. In V1aR overexpressing muscles, the expression levels of phospho-FoxO3a and phospho-Akt is increased compared with TNF alone, while the native Foxo3a is reduced. (b–d) Densitometric analysis of three independent experiments of phospho-Akt, phospho-FoxO3a, and native FoxO3a expression levels. (e) Real-time PCR analysis revealed that the strong upregulation of atrogin-1 expression observed in the sample overexpressing TNF alone is downregulated in V1aR+TNF-transfected muscles. *P < 0.05; **P < 0.01 by Student's *t*-test.

TNF is known to induce protein degradation as a result of activation of the ubiquitin-dependent proteasome pathway [7]. On the other hand, the PI3K/Akt signaling is one of the most critical pathways involved in the regulation of skeletal muscle mass [8, 12]. Akt phosphorylates FoxO transcription factors in multiple sites, leading to the exclusion of phosphorylated FoxO proteins from the nucleus and inhibition of atrogin-1 expression [47]. We therefore analyzed the effect of TNF on Akt and FoxO3a expression in the presence and absence of V1aR overexpression. Here we demonstrate that, in muscle overexpressing TNF, the phosphorylated form of Akt is downregulated compared with mock-transfected muscle, while the expression of native FoxO (FoxO3a) is high. The double band of native FoxO3a is ascribable to the presence of posttranslational modifications of FoxO3a, such as acetylation or ubiquitination. By contrast, phospho-Akt is upregulated in V1aR overexpressing muscles, either alone or in combination with a high TNF level (Figure 7(a)). Indeed, phospho-FoxO3a is downregulated only in samples overexpressing TNF alone, and the expression of atrogin-1 in this condition is strongly upregulated (Figures 7(a) and 7(e)). These results demonstrate that V1aR overexpression stimulates the PI3K/Akt pathways leading to phosphorylation of FoxO transcription factors, which in turn results in downregulation of atrogin-1 expression.

## 4. Discussion

In this study, we show that the negative effects of TNF on muscle regeneration and inflammation are strongly counteracted by stimulation of AVP signaling. AVP, a neurohypophyseal nonapeptide, is a potent myogenesis-promoting factor both* in vitro* and* in vivo*. By interacting with V1aR, AVP increases cytosolic Ca^2+^ concentrations, upregulates Myf-5 and myogenin expression, and activates Ca2^+^/calmodulin-dependent protein kinase (CaMK) and CnA signaling pathways [16, 17, 48–51]. Moreover, local V1aR overexpression results in acceleration of the regeneration process, as demonstrated by rapid resolution of inflammation, earlier activation and fusion of satellite cells, and formation of regenerating fibers compared with the mock-transfected muscles [21].

To better clarify the molecular pathways involved in the positive effects of V1aR overexpression in muscle homeostasis, we induced morphological alterations in TA muscles by local overexpression of TNF and analyzed the effects of high levels of V1aR on TNF-induced muscle atrophy.

TNF is a proinflammatory cytokine known to induce murine myoblast apoptosis [52] and block human muscle satellite cell differentiation [53]. Chronic exposure to low levels of circulating TNF inhibits muscle regeneration and induces cachexia [35, 54].

Morphological analysis clearly demonstrated that the accumulation of infiltrating cells in muscle overexpressing TNF alone dramatically decreases when TNF and V1aR are overexpressed together. Moreover, while V1aR does not significantly modify the number of regenerating fibers compared with samples overexpressing TNF alone, it does affect the fiber size distribution suggesting that it may play a role in acceleration of the regeneration process. Furthermore, a comparison between mock and V1aR-transfected muscles indicated that V1aR overexpression exerts a hypertrophic effect on the fibers, regardless of the regeneration process, which may be attributable to either increased fusion of satellite cells or increased synthesis and accumulation of contractile proteins. Inflammation is a critical component of muscle physiology that represents an important phase in regeneration and is often associated with severe and progressive fibrosis [3, 36, 37, 55]. Invasion by neutrophils and macrophages characterizes the initial phases of inflammation. This is followed by downregulation of the inflammatory response, thus preventing further damage and favouring regeneration [56]. The shift from the M1-macrophage-dependent necrotic environment to the M2-macrophage phase characterized by stem cell recruitment and differentiation is important insofar as it promotes regeneration [57, 58]. This could also explain the slight increase in CD163 (involved in anti-inflammatory responses) expression occurring in TNF overexpressing muscles [2, 3]. Our data are in line with recent findings demonstrating that the V1aR agonist dramatically reduces the mRNA level of proinflammatory cytokines in astrocytes [59].

The enhanced regenerative capacity of V1aR overexpressing muscles correlated with dampening of inflammation and connective tissue accumulation; consequently, fibrosis and prolonged infiltration by monocytes/macrophages typical of TNF overexpressing muscle were more rapidly resolved in the presence of high levels of V1aR. This finding is supported by the V1aR-dependent modulation of NF-*κ*B expression, one of the most important molecular players in the activation and maintenance of inflammation in response to TNF stimuli [9, 60].

Local overexpression of V1aR selectively downregulated the proinflammatory cytokines, CCL2, IL1*β*, IL-6, whose expression is associated with sustained mononuclear cell influx and the switch from acute to chronic inflammatory process [3, 5]. Interestingly, V1aR upregulated the expression of anti-inflammatory cytokines, including CD163, IL-10, and IL-4, which play a major role in promoting growth and regeneration [61]. In particular, several works have identified IL-4 as a key cytokine in myogenic fusion processes. Being directly correlated with CnA activity, IL-4 is synthesized and secreted after the translocation of nuclear factor of activated T-cells into the nucleus and stimulates fusion of myoblasts to preformed myotubes [44–46]. Moreover, we previously demonstrated that IL-4 represents a key cytokine in the mediation of the effects of AVP on skeletal muscle homeostasis [21]. In the present study we found that TNF stimulates satellite cell activation and muscle regeneration, as demonstrated by the upregulation of Pax7 and desmin expression, but, as expected, impinges on the maturation process as shown by the low levels of the late differentiation marker MHC. By contrast, the overexpression of V1aR counteracted the negative effects of TNF, stimulating muscle growth and maturation as demonstrated by the increased expression levels of myogenin and MHC in TNF+V1aR overexpressing muscles.

One of the most important mechanisms controlling cellular and protein turnover is mediated by Akt-FoxO. A reduction in the activity of the Akt pathway, as observed in different models of muscular atrophy, results in decreased levels of phosphorylated FoxO and consequent upregulation of atrophy related genes [14, 62], which are responsible for increased protein degradation through the ubiquitin-proteasome system [7, 63, 64]. Here we demonstrate that TNF overexpression upregulates dephosphorylated FoxO expression, thus promoting the transcriptional activation of atrogin-1. By contrast, V1aR overexpression stimulated the PI3K/Akt pathways, leading to phosphorylation of FoxO transcription factors and resulting in the inhibition of atrogin-1 expression.

Since neurohypophyseal hormones are not canonical regulators of skeletal muscle structure and function, the physiological relevance of our findings merits discussion. A large body of evidence indicates that AVP and/or OT play a significant role in promoting differentiation and hypertrophy of myogenic cells in culture. Recently, Breton et al. provided evidence that functional oxytocin receptors are present in human primary myoblasts [19]. The finding that levels of immunoreactive AVP, which are high in embryonic skeletal muscle, decline during gestation and reach very low levels at birth points to a role of AVP in muscle development [65]. These data, combined with our results demonstrating a modulation of V1aR endogenous expression during the regeneration process, suggest that AVP signaling plays a significant role in skeletal muscle homeostasis [21]. Interestingly, several authors have shown that exercise, a physiological hypertrophic cue, significantly increases circulating AVP, both in humans and in other mammals, thus posing the theoretical basis for the physiological regulation of muscle hypertrophy by neurohypophyseal hormones [49, 66–69]. De Jager et al. recently reported that treatment of cattle with anabolic steroids unexpectedly led to a high expression of mRNA encoding oxytocin in muscle, accompanied by a high level of circulating oxytocin in the plasma [70], suggesting that OT is involved in mediating the anabolic effects of the treatment.

## 5. Conclusions

Our findings show that the stimulation of AVP signaling in muscle enhances the regeneration process by attenuating inflammation and fibrosis and by modulating protein degradation. Stimulation of AVP signaling might represent an interesting novel strategy to counteract muscle decline in aging or in muscular pathologies.
